# Navigating the Unique Challenges of Caregiving for Children with Rare Diseases: Are the Care Experiences of All Caregivers the Same? A Focus on Life-Limiting Rare Diseases

**DOI:** 10.3390/jcm13154510

**Published:** 2024-08-01

**Authors:** Dariusz Walkowiak, Jan Domaradzki, Renata Mozrzymas, Łukasz Kałużny, Jarosław Walkowiak

**Affiliations:** 1 Department of Organization and Management in Health Care, Poznan University of Medical Sciences, 60-356 Poznań, Poland; 2 Department of Social Sciences and Humanities, Poznan University of Medical Sciences, 60-356 Poznań, Poland; jandomar@ump.edu.pl; 3 Research and Development Center, Regional Specialist Hospital, 53-413 Wrocław, Poland; 4 Department of Pediatric Gastroenterology and Metabolic Diseases, Poznan University of Medical Sciences, 60-356 Poznań, Poland

**Keywords:** rare diseases, caregivers, children with rare diseases, phenylketonuria, PKU, life-limiting rare diseases

## Abstract

**Background**: Caregiving experiences in rare diseases (RDs) vary based on factors such as specific clinical entity, disease severity, the child’s age, and available support and resources, leading to challenges that significantly impact caregivers’ lives. This study investigates whether caregivers of children with different RDs encounter varied aspects of care. **Methods**: This study was conducted as a self-administered, anonymous, computer-assisted online survey, focusing on the challenges of caregiving for children with RDs. Questions covered aspects such as information availability on RDs, diagnostic processes, modern treatment accessibility, family physicians and specialists, the impact of caregiving on personal life, family dynamics, and financial challenges. To achieve our study objectives, we categorized caregivers of children with RDs into two groups to compare various aspects of caregiving: caregivers of children with phenylketonuria (PKU) (*n* = 175) and those caring for children with life-limiting rare diseases (LLRD) (*n* = 226). **Results:** Caregivers of children with LLRD reported greater emotional challenges, personal sacrifices, and financial burdens compared to caregivers of children with PKU. Significant differences included heightened emotional distress, more frequent conflicts, and lower assessments of healthcare support among LLRD caregivers. Although family support ratings were similar between the groups, perceptions of financial concerns and interactions with the healthcare system varied significantly. **Conclusions**: This study, representing the inaugural systematic comparison of specific caregiver cohorts overseeing children with RDs across a substantial sample size, provides valuable insights. The findings lay a crucial foundation for precisely tailoring assistance and support initiatives to meet the unique needs of caregivers facing various RDs in diverse contexts.

## 1. Introduction

In the absence of a universally accepted global definition for rare diseases(RDs), the European Commission characterizes them as comprising 5000 to 8000 life-threatening or chronically debilitating conditions, collectively impacting 27 to 36 million individuals within the European Union, with each disease affecting no more than 5 in 10,000 [1,2]. The pervasive lack of effective treatments and precise diagnostic methods for the majority of RDs underscores a substantial unmet medical need, posing a formidable challenge to public health [3,4]. RDs, often severe and chronically debilitating, impose a significant burden not only on the affected individuals but also on their families [5,6]. Many RDs manifest early in life, resulting in a curtailed lifespan and necessitating lifelong care. Approximately 80% of RDs are of genetic origin. On average, patients endure a five-year waiting period for a diagnosis, and even upon diagnosis, treatments are available for only 6% of recognized RDs [7]. Beyond the realm of physical health, individuals with RDs bear a disproportionate load of psychological, financial, and social challenges [1,8]. Caregiving for children with RDs is full of challenges, including a scarcity of accessible and reliable information and expertise, which leaves caregivers in constant uncertainty [9,10].

RDs form a diverse and extensive category of conditions that require extended care, presenting substantial challenges on both the public and social fronts [11,12]. These conditions substantially impact the lives of caregivers who may perceive impairments in their professional, social, and family spheres, resulting in diminished physical and emotional well-being [13,14]. One pivotal challenge confronted by caregivers is the often-delayed or misdiagnosed nature of RDs, prolonging the time before appropriate medical interventions can commence [15,16,17]. This engenders frustration and helplessness among caregivers, compounded by the emotional toll of witnessing a child grappling with the challenges of a rare disease, leading to stress, anxiety, depression, and an overarching sense of helplessness [13,18,19,20].

Financial implications form a critical dimension of caregiving for children with RDs. The substantial costs associated with medical expenses, specialized treatments, and ongoing therapies may not be fully covered by insurance, imposing financial strain on caregivers [21,22,23]. The rarity of these diseases hampers connections with others who share similar experiences, fostering feelings of isolation and misunderstanding. Unique educational needs present an additional layer of complexity, compelling caregivers to navigate intricate educational systems to secure appropriate accommodations and support for their children. Caregiving responsibilities, involving the management of complex medical regimens, medication administration, and provision of physical assistance, often result in physical exhaustion for caregivers [24,25,26].

The coordination of care among various specialists, management of multiple healthcare providers, and navigation of bureaucratic healthcare systems pose additional hurdles for caregivers [27,28]. Facing overwhelming caregiving demands, stress, and negative impacts on psychological health and family functioning, caregivers of children with RDs navigate a challenging landscape that may strain family dynamics, potentially leading to siblings feeling neglected. In the role of advocates, caregivers actively seek appropriate medical care, engage with researchers, and participate in advocacy efforts to raise awareness about RDs. Addressing these intricately interconnected challenges necessitates a holistic approach encompassing medical, social, and psychological support services, striving to provide comprehensive assistance to caregivers of children with RDs [6,23,24,29,30].

Caregiving experiences vary based on specific RDs, the severity of the disease, the age of the child, and the availability of support and resources. Caregivers grapple with challenges that negatively impact their quality of life, including limited access to quality healthcare, a shortage of experienced healthcare professionals, and a lack of knowledge about the diseases they are dealing with [31,32,33,34]. The burden of caring for children with complex medical needs is significantly greater than is generally understood by both multidisciplinary healthcare teams and the general public [6]. There have been suggestions that parents of children with rare genetic syndromes experience greater distress compared to parents of children with Down syndrome or intellectual disabilities of unknown etiology [35].

The concept of RDs is expansive, encompassing several thousand distinct conditions. Often referred to as an “umbrella” term, it connects these diseases by their shared characteristic of rarity. However, many initiatives targeting RDs and their caregivers treat them as if they are dealing with a uniform group. In reality, the number and variety of these diseases suggest otherwise. Given that RDs are, by definition, life-threatening or chronically debilitating conditions, it is crucial to consider whether the diseases themselves and the associated risks affect the functioning of caregivers of children with RDs. A life-limiting disease is characterized by a condition that typically results in premature death, such as Duchenne muscular dystrophy or cystic fibrosis [36]. Conversely, a life-threatening disease is one where premature death is highly probable, yet survival into adulthood remains a possibility. This category includes children undergoing active oncological therapy or those receiving intensive care following acute trauma [36]. Similarly, the concept of life-threatening conditions, according to other authors, also encompasses substantial prematurity, critical congenital heart disease, cancer, or conditions resulting in severe neurologic impairment [37]. Additionally, certain groups of conditions are identified as potentially leading to palliative care for children and young people, inherently related to the expected or possible death of the patient [38,39]. There are also concepts of categorizing pediatric palliative care patients into groups that represent the four most common illness trajectories, regardless of the type of disease [40]. However, this approach means that conditions such as Duchenne muscular dystrophy, glioblastoma, neuroblastoma, and spinal muscular atrophy are included in one of these illness trajectories. Additionally, the literature features very general divisions of life-limiting and life-threatening conditions, such as separating them into congenital and cancer groups [41]. As a result, RDs are somewhat outside the mainstream of research on life-threatening conditions, appearing in some studies but never being a separate research group.

This article aims to compare the differences in the experiences of parenting a child with life-threatening RDs and those with less severe conditions, exemplified by phenylketonuria (PKU). PKU was chosen as it represents a paradigmatic example of effective therapeutic measures available for RDs: Poland offers diagnostic tests for early detection of PKU, and national screening programs for newborns enable early dietary intervention to prevent mental retardation. This is particularly important in countries like Poland, which have yet to fully implement national plans for RDs and still face many institutional barriers in accessing modern diagnostics using large-scale genomic testing, medications, and high-quality, innovative healthcare services and diets to manage the specific nutritional needs of RDs.

## 2. Materials and Methods

This study utilized data from a broader project focused on understanding the challenges, needs, and emotional experiences of parents caring for children with rare diseases (CRD) [42,43]. The research was carried out from October 2022 to May 2023, involving individuals who volunteered and participated in the study. Given the absence of a registry for pediatric rare disease patients in Poland and the unknown exact number of CRD cases, the study was designed as a self-administered, anonymous, computer-assisted online survey. Study participants were recruited through convenience sampling, with the support of various rare disease foundations, patients’ associations, and organizations via their web pages and Facebook, along with physicians specializing in treating RDs. Inclusion criteria encompassed individuals who were parents or family members providing care for CRD aged 0 to 18, capable of using electronic devices, and participating in online surveys. Caregivers utilized electronic devices (e.g., computers, tablets, or smartphones) to complete the survey, which took approximately 15–20 min. Two follow-up messages were sent in January and March. All caregivers provided written informed consent, and ethical approval was obtained from the Poznan University of Medical Sciences Bioethics Committee (KB-833/22, 22 October 2022).

The reported data stem from an original closed-ended questionnaire developed after a comprehensive literature analysis. This research delves into the experiences of Polish caregivers, focusing on the challenges of caregiving for children with RDs. The selected questions focused on caregivers’ experiences with the healthcare system, including the diagnostic process, availability of information on RDs, access to family physicians and specialists, accessibility to modern treatments for RDs, including drugs and rehabilitation, availability of psychological support for CRD and parents, contact with a genetic clinic, and support from physicians. Health status and quality of life instruments frequently lose their validity when applied outside the context for which they were originally developed [44]. Given the absence of a specific tool for assessing CRD caregivers’ experiences with healthcare in Poland, an ad hoc questionnaire was constructed following the guidelines of the European Statistical System [45]. Additionally, the questionnaire addressed issues related to caregivers’ functioning, well-being, the impact of caregiving on personal life, financial challenges, and family and friendship relationships. After evaluation by a pediatrician specializing in RDs, a public health specialist, and a medical sociologist, the questionnaire underwent pre-testing on an online platform with 10 caregivers. Following this, it was reevaluated by additional medical specialists, leading to the re-formulation of six questions. The survey was revised based on the responses received. Participants were informed about the study’s purpose, its voluntary and anonymous nature, and its confidentiality. They were also given the option to end the interview at any time and to withhold personal information due to the sensitive nature of the topic. The survey was placed on an internet platform and electronically distributed to each caregiver after informed consent was received from all volunteers included in the study. All themes were explored using closed-ended questions on a 5-Likert scale ranging from 1 (strong dissatisfaction or disagreement) to 5 (high satisfaction or agreement).

In health-related research, internal consistency reliability is employed to assess the reliability of health-related measures or quality-of-life scales. This ensures that the measures consistently assess symptoms, treatment effects, or health-related outcomes. Validity refers to the degree to which a measure accurately assesses the specific concept, trait, or construct it claims to assess, indicating the truthfulness of the measure. The internal consistency of our questionnaire was evaluated using Cronbach’s Alpha and McDonald’s Omega [46,47,48], both demonstrating high levels of internal consistency with Cronbach’s Alpha = 0.901, 95%CI [0.886–0.915] and McDonald’s Omega = 0.898, 95%CI [0.884–0.913] [49,50].

To achieve our study objectives, we categorized caregivers of CRDs into two groups, with the aim of comparing various aspects of caregiving to highlight differences in perspectives resulting from the severity of the disease, the method of diagnosis, and the available therapies. Through survey analysis, we sought to establish homogeneous caregiver groups. Addressing diverse patient groups, ranging from 175 in the case of phenylketonuria (PKU) to varying sizes in other rare and ultra/hyper-rare diseases, posed a challenge. The analysis outcomes led to the decision to differentiate two groups: one entirely uniform, consisting solely of PKU caregivers, and the other composed of caregivers dealing with diseases that either prematurely end the patient’s life or pose a life-threatening risk. This consideration takes into account the caregiver’s role in care and their emotional experience. This distinction aligns with existing literature, where all life-limiting diseases, not solely rare ones, are classified as such [40,51,52,53,54]. For the purposes of our study, we categorized all diseases resulting in the premature death of a child or posing a risk of premature death as Life-Limiting Rare Diseases (LLRDs).

A total of 226 caregivers were identified, providing care to children with 52 different LLRDs. These encompassed diseases with a higher prevalence, such as Dravet syndrome, Cri du Chat syndrome, Mucopolysaccharidosis, 3-hydroxy acyl-CoA dehydrogenase deficiency of long-chain fatty acids, or deficiency of medium-chain acyl-CoA dehydrogenase. A notable portion of the group consisted of caregivers for children who were the sole participants with their condition in our study, such as those with Citrullinemia type 1, Duchenne muscular dystrophy, Propionic aciduria, Glutaric aciduria I, Tyrosinemia I, Tay–Sachs disease, or Niemann–Pick disease. The qualification process for the study was conducted by two doctors with over 25 years of experience in treating RDs (Ł.K. and J.W.). Selection into the group was performed on an individual, case-by-case basis and was not solely based on assumptions about the prognosis of a given disease. In cases where doubts arose about the prognosis, individuals were not included in the research group. Consequently, the final analysis included a total of 401 caregivers whose responses were taken into account (175 PKU, 226 LLRD).

Statistical analysis was conducted using JASP 0.18.3, with a significance level set at 0.05. Differences in assessments of various aspects of care were evaluated using the Mann-Whitney test, while differences in the assessment of the child’s health problems were analyzed using the chi-square test. The application of network analysis has witnessed a rise in exploratory investigations into psychological behavior, representing a departure from the traditional perspective where latent variables explain correlations among variables, and observed variables are assumed to causally influence one another. In many scientific fields, researchers study phenomena best characterized at the systems level. To understand such phenomena, it is often insufficient to focus solely on the operation of individual system components. Instead, it is crucial to examine how these components are organized and interact, which can be represented in a network. The advent of network science has underscored the value of this approach, providing significant insights into a wide range of scientific phenomena. Network analysis offers a unique perspective on interconnected systems, enabling researchers and analysts to uncover hidden patterns and relationships that might not be apparent through traditional analytical methods. This approach facilitates the integration of diverse data, visualizes their interactions or relationships, and allows for inferences within the context of sociological mechanisms [55,56].

The network was constructed based on partial correlations among variables, enabling the identification of distinct interactions that might go unnoticed in multiple regression analysis. This study encompasses 25 nodes/items, and the strengths of associations between nodes were determined using Pearson correlation analyses, with thicker edges denoting more robust relationships. For the two groups, independent estimates of network models were generated using sparse Graphical Gaussian Models (GGM) alongside a graphical least absolute shrinkage and selection operator (LASSO) method. The model selection process was guided by the Extended Bayesian Information Criterion (EBIC). To produce easily interpretable networks with uniform edge lengths and prevent visual obstructions from overlapping edges, the model employed the force-directed Fruchterman-Reingold algorithm [57]. The tuning parameter of the EBIC, crucial for balancing the inclusion of false edges and the elimination of true edges, was set to 0.5 in accordance with the recommendations of Foygel and Drton [58]. The network analysis was performed using JASP version 17.1, leveraging the R package qgraph with the “EBICglasso” estimation method (https://jasp-stats.org/; accessed on 10 February 2023).

## 3. Results

Table 1 provides a concise overview of two caregiver groups: those caring for children with LLRD and those caring for children with PKU. It includes key information about caregiver demographics, their relationship to the child, and an assessment of the children’s health conditions. Females constituted a majority of the caregivers in both groups, 94.2% in the LLRD group and 89.1% in the PKU group. Caregiver ages in the LLRD group range from 19 to 57 years, with a median age of 37. Most caregivers in the LLRD group are mothers (92.4%). In the PKU group, the majority are also mothers (88.6%). Children’s ages in the LLRD group range from 0.2 to 18 years, with a median age of 7. In the PKU group, children’s ages range from 0.1 to 18 years, with a median age of 6.

Table 2 compares the ratings given by caregivers of LLRD and PKU children on their perceived severity of health problems. In the LLRD caregiver group, a majority (54.4%) perceive their child’s health problems as “very severe,” followed by “severe” (21.2%) and “moderate” (19.5%). In contrast, the PKU caregiver group exhibits a different distribution, with “moderate” and “mild” health problems as the most prevalent categories.

The analysis of Table 3 provides valuable insights into the emotional and practical challenges faced by caregivers of children with LLRD when compared to those caring for PKU children. LLRD caregivers reported significantly higher levels of emotional control problems, a sense of shame, nervousness/impulsivity, emotional lability, and impatience/irritation compared to PKU caregivers (*p* < 0.001). Moreover, LLRD caregivers, in contrast to PKU caregivers, more frequently sacrificed their passions, hobbies, and plans due to their caregiving role (*p* < 0.001). The data also reveal that caregivers of LLRD children encounter more conflicts and difficulties (*p* < 0.001), particularly in balancing their own needs with those of their children (*p* < 0.001). LLRD caregivers rated support from physicians, physicians’ empathy, access to financial help with rehabilitation, support from healthcare professionals, and physicians’ practical information about RD lower than PKU caregivers (*p* < 0.001). However, there were no significant differences in the level of support received from family for both groups. Finally, LLRD caregivers reported more conflict in their families (*p* < 0.001), a heavier burden concerning the cost of medicines and medical care (*p* < 0.05), and problems with the reimbursement or purchase of medicines compared to PKU caregivers (*p* < 0.05).

**Figure 1:**
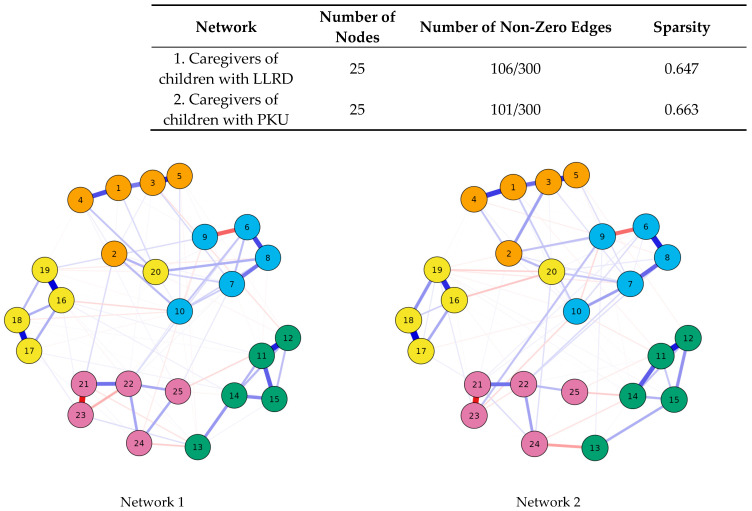
Network analysis of caregiving experiences. 1–5 Impact on Personal Life; 6–10 Conflict and Fulfillment of Needs; 11–15 Physician and Healthcare Support; 16–20 Family Dynamics and Social Support; 21–25 Financial Challenges and Medication Concerns.

The network analysis (Figure 1) involves two separate networks: Network 1, which represents caregivers of children with LLRD, and Network 2, which represents caregivers of children with PKU. Each network consists of 25 nodes, which represent 25 items from Table 2. Blue lines signify positive associations, whereas red lines denote negative ones. The strength of association is conveyed through both the width and brightness of the edges. The number of non-zero edges in each network indicates the connections or relationships between items. In Network 1, there are 106 non-zero edges out of a possible 300, and in Network 2, there are 101 non-zero edges out of 300. These edges may represent interactions, associations, or shared characteristics between items. Network 1 has a sparsity of 0.647, while Network 2 has a sparsity of 0.663. A lower sparsity value indicates a denser network with more connections. The variations in both networks suggest differences not only in the intensity of individual items but also differences in the formation of interactions between items in both groups.

## 4. Discussion

To our knowledge, our study represents the first systematic attempt to compare distinct cohorts of caregivers overseeing children with RDs across a substantial sample size. The findings align closely with expectations, showing that caregivers managing different RDs encounter unique challenges alongside common experiences. These identified differences, as well as areas of similarity, offer valuable insights. They form a pivotal foundation for more precisely tailoring assistance and support initiatives to address the diverse needs of caregivers in various contexts. Our results support previous research [59], underscoring the importance of identifying RDs that impose greater burdens and potentially exacerbate caregiver health. These insights are crucial for informing socio-health policies aimed at improving precision in support measures. This study signals the potential beginning of more targeted health policies and state aid for specific vulnerable groups and their caregivers.

Research on the experiences of caregivers of children with different RDs reveals common challenges, such as the impact on relationships and worries [60], reduced quality of life [61], increasing levels of anxiety and depression [62], and distress related to diagnosis, care, and societal responses [63]. Navigating the healthcare system is a significant challenge, with parents often feeling isolated and burdened with the role of care coordinator [31]. Despite these challenges, parents find support in self-help groups, peer support, and health professionals [31,60,63]. These findings underscore the need for interventions and support tailored to the specific needs of caregivers of children with RDs. Our results show that various aspects of caregiving may be experienced differently by caregivers of children with different diseases, emphasizing the real need to individualize the support and assistance provided to caregivers. Caregivers of children with LLRD often grapple with a myriad of emotions and face unique challenges. These caregivers commonly experience heightened emotional distress, including emotional control problems, a sense of shame, nervousness/impulsivity, emotional lability, and impatience/irritation. The burden is not only emotional but extends to practical sacrifices, as caregivers of LLRD children frequently find themselves giving up their passions, hobbies, and plans due to the demands of the caregiving role.

Conventional epidemiological analyses, such as regression, typically examine the association between an exposure and an outcome as a one-to-one correspondence. This approach has a significant limitation, often described as the “black-box” nature of the analysis, because it cannot fully elucidate complex relationships such as biological pathways or sociological dynamics. To address these hidden mechanisms, network analysis has emerged as a new framework [64]. A system may be defined as a complex of interacting components and the relationships among them, which allow for the identification of a boundary-maintaining entity or process through systems theories. From a network perspective, health behaviors and outcomes can be conceptualized as emergent phenomena resulting from a system of reciprocal interactions. Network analysis provides a powerful methodological approach to investigate these intricate patterns, offering a deeper understanding of the systems in which they occur [56]. The variations in both networks indicate differences not only in the intensity of individual items, as shown in Table 3, but also in how interactions between items are formed in each group. In the conducted network analysis of caregivers of children with LLRD, there are a greater number of non-zero edges, and some observed non-zero edges differ from those in caregivers of children with PKU. For example, the relationship between items 6 and 10 (questions regarding giving up passions, hobbies, and plans of the caregiver and the feeling that the caregiver’s needs are unimportant to others) is essentially nonexistent for caregivers of children with PKU. The same goes for items 24 and 25. This indicates not only different perceptions of individual assessments in different groups but also, at least to some extent, different ways of mutual relations between different items.

RDs often involve life-limiting conditions, demanding continuous caregiving [23,54]. The course of the disease follows specific stages, and the lives of caregivers undergo specific phases accordingly, as identified in previous research [65]. This is why the implementation of an adequate, comprehensive palliative care framework in RDs, vital in order to ensure effective support for patients and their families [54], could be designed in accordance with those stages to respond to the needs of patients and caregivers at each stage. However, ratings for support and contacts with the healthcare system in our study were notably lower for caregivers of LLRD children. This state of affairs could potentially be improved.

Financial challenges do not significantly differentiate the study group in general terms, but specific areas, such as drug costs, do show significant differences. This may imply that, to a lesser extent than other circumstances, the child’s disease imposes a substantial burden on the family. Previous research has indicated that, upon diagnosis, one parent, typically the mother, often leaves employment to dedicate themselves to the child’s care [59,62,66]. Managing a family with a sick child on a single income is usually a considerable challenge, especially given the expenses related to rehabilitation, specialized diets, and medications. From this perspective, financial concerns in families with a sick child may be less specific to the disease and more a consequence of inadequate income and the necessity to cover additional expenses. However, it is worth noting that the burden on caregivers with LLRD may be higher than in the group of caregivers of children with PKU, as suggested by their responses regarding the cost of medicines and medical care as a source of significant burden.

Caregivers of children with various RDs often face significant disruptions to their daily routines [24,67,68,69,70]. The level of impact can vary depending on factors such as the severity of the disease, the need for constant medical supervision, and the presence of associated disabilities or complications.

Caregiving for a child with an RD can take a toll on caregivers’ emotional well-being [13,51,54,62,71]. Feelings of stress, anxiety, depression, and isolation are commonly reported across different conditions. Coping mechanisms and support networks play crucial roles in mitigating these challenges. The accessibility and availability of healthcare services vary among different RDs, with caregivers often encountering barriers such as limited access to specialized medical facilities, extended wait times for appointments, and difficulties in obtaining timely diagnoses and treatment [24,63,67,72]. Balancing their own needs with those of their children becomes a source of conflict and difficulty for caregivers of LLRD children. This struggle is reflected in their assessments of physicians’ empathy, access to financial help with rehabilitation, support from healthcare professionals, and physicians’ practical information about RD, all of which are rated lower compared to caregivers of children with PKU. Moreover, this same group evaluates the support received from physicians significantly worse, suggesting a systemic issue where a subset of caregivers in need of special support may not be receiving it. Considering that the majority of children in this group are diagnosed based on symptoms, and only a small portion through neonatal cases, such as in PKU, a concerning picture emerges of the challenges faced by caregivers. It is possible that these challenges contribute to higher instances of emotional control problems and emotional liability, as well as more frequent experiences of impatience and irritation.

The financial implications of caregiving vary depending on factors such as the need for specialized equipment, medications, therapies, and frequent hospitalizations. Caregivers may struggle with managing medical expenses, navigating insurance coverage, and balancing caregiving responsibilities with work obligations. Caregivers often express the need for accurate and up-to-date information about their child’s condition, treatment options, and available support services. Access to reliable educational resources and opportunities for peer-to-peer networking can empower caregivers and enhance their ability to advocate for their child’s needs [33,54,73,74]. Creating visibility for caregivers of patients with RDs involves recognizing their valuable contributions to economies, healthcare systems, and society. Despite their critical role, this group lacks formal recognition and support in key areas: inadequate support programs to ease caregiving responsibilities, limited financial assistance for caregiving costs, and insufficient workplace accommodations to alleviate time and financial strain [25].

Caring for a child with a rare disease can significantly influence family dynamics and relationships. Siblings may require additional attention and support, and parents may experience strain in their marital or partner relationships [37,52,59,75]. Maintaining open communication and mutual support within the family unit is crucial for resilience and cohesion. In both study groups, no significant difference was found regarding practical help from family or friends, as well as emotional support from their family. However, it is regrettable that in both groups, this support was similar but not very high. Only some caregivers can consistently rely on such support, either always or often. In addition to these emotional and practical challenges, caregivers of LLRD children often face strained family dynamics. They may experience conflicts within their families and perceive a heavier burden concerning the cost of medicines and medical care. Financial concerns are heightened, with potential problems arising in the reimbursement or purchase of medicines. These caregivers navigate a complex landscape of emotional, practical, and financial difficulties, highlighting the need for targeted support and understanding within this specific caregiving context.

Despite the many challenges they face, caregivers often demonstrate remarkable resilience and resourcefulness in navigating the complexities of RDs. Finding meaning, purpose, and moments of joy in caregiving experiences can foster a sense of hope and optimism amid adversity [53,72,76]. While these themes provide a broad framework for understanding the caregiver experience across different RDs, it is essential to recognize the nuances and specificities of each condition. Caregivers’ needs, preferences, and coping strategies may vary widely, highlighting the importance of tailored support interventions and holistic approaches to care.

While this study provides an initial exploration of the caregiving experiences of parents with life-limiting rare diseases in Poland, it has inherent limitations. Even though the questionnaire used in this survey was reviewed by external experts in pediatrics, public health, and medical sociology and was pre-tested in a pilot study with RD caregivers, it was not formally validated. Despite a reasonably high response rate, the estimated number of children with RDs in the country surpasses the participants in this study. Consequently, these findings exclusively reflect the perspectives of participating parents and should not be generalized to the entire population of Polish caregivers for CRD, as not all caregivers are affiliated with the online support groups, associations, or organizations that assisted in the recruitment process. Moreover, due to non-participation or reluctance to disclose personal information, the survey only captures the viewpoints of parents who chose to engage in the study and share their experiences. Despite our professional approach, it is possible that not all selections for the LLRD group were entirely accurate, relying solely on completed questionnaires rather than medical interviews. This approach was necessary to ensure the anonymity of caregivers expressing their opinions on the study’s subject.

The recruitment approach also poses a limitation; although the first National Plan for Rare Diseases was adopted in Poland in 2021, there is still no registry of individuals with RDs, and the exact number of pediatric RD patients is unknown. Furthermore, none of the RD foundations, patient associations, or organizations involved in the study were able to provide specific numbers of pediatric RD patients for any given condition.

Another limitation may be the overwhelming majority of responses given by female CRD caregivers, most often mothers. This is likely a result of their primary caregiving roles. However, the small number of male responses should be considered a limitation, as it provides a one-sided perspective, even though the majority of CRD have more than one caregiver.

An important, albeit positive, limitation of our article is the ongoing development in medicine, particularly the advent of effective therapies, new drugs, and gene therapy. A disease that currently leads to premature death may become completely or significantly curable in a few years.

## 5. Conclusions

In conclusion, the two study groups differ in their self-assessments of various aspects of care. Caregivers of children with LLRD, in most cases, assessed these aspects differently compared to caregivers of children with PKU. These differences encompassed both the Impact on Personal Life and Conflict and Fulfillment of Needs categories, as well as the Physician and Healthcare Support categories. Caregivers of children with LLRD more frequently reported emotional control problems, nervousness/impulsivity, emotional lability, and impatience/irritation. They also expressed a higher frequency of conflicts between their own needs and those of their RD child, as well as feeling that the role of caregiver forced them to give up their own passions, hobbies, and plans. Additionally, caregivers of children with LLRD rated physicians’ empathy, support for RD children and caregivers from healthcare professionals, physicians’ practical information about RD, and support caregivers receive from physicians lower. Both groups similarly rated support and help from their family and relatives/friends. Although a minority, caregivers of children with LLRD experienced more conflicts in their families than did caregivers of children with PKU. Financial challenges partially differentiated the studied groups, particularly in terms of contact with the healthcare system. However, worries about the family’s finances and assessing the financial situation were similar for both groups.

Our study has shown that caregivers of children with LLRD face significantly greater burdens than those with other RDs. Consequently, it may be worth considering implementing strategies that have proven effective in managing PKU, such as screening programs or, at the very least, improving access to modern molecular diagnostics. Additionally, organizing the care system around regional or national highly specialized centers is crucial. Currently, every patient with PKU in Poland receives care from one of nine hospitals with specialists in metabolic pediatrics, whereas a patient with Dravet Syndrome does not have access to such organized care post-diagnosis. Furthermore, there is a need to enhance access to modern therapies and rehabilitation, as well as psychological care and financial support for caregivers. Some of these initiatives were included in the National Plan for Rare Diseases, and it is now time to implement them fully.

## Figures and Tables

**Table 1:** Characteristic of caregivers and children in LLRD and PKU groups.

Characteristics	LLRD Children’s Caregivers *n* (%) *n* = 226	PKU Children’s Caregivers *n* (%) *n* = 175
Caregiver’s sex		
female	213 (94.2)	156 (89.1)
male	13 (5.8)	19 (10.9)
Caregiver’s age		
Range	19–57	23–72
Median	37	37
IQR (1–3)	33–41.5	34–42
Mean (95%CI)	37.4 (36.6–38.2)	37.8 (36.8–38.7)
SD (95%CI)	5.9 (5.3–6.5)	6.4 (5.3–7.6)
Relationship with RDs child		
mother	209 (92.4)	156 (88.6)
father	13 (5.8)	17 (9.7)
grandparent	2 (0.9)	2 (1.1)
spouse	2 (0.9)	0 (0)
legal guardian	0 (0)	1 (0.6)
Child’s age M (SD)		
Range	0.2–18	0.1–18
Median	7	6
IQR (1–3)	4–11	3.25–10
Mean (95%CI)	7.8 (7.1–8.4)	6.6 (5.9–7.2)
SD (95%CI)	4.7 (4.3–5)	4.4 (3.9–4.7)

**Table 2:** Perceived Severity of Children’s Health Problems.

How Would You Rate Your Child’s Health Problems	LLRD Children’s Caregivers *n* (%) *n* = 226	PKU Children’s Caregivers *n* (%) *n* = 175	*p*
very severe	123 (54.4)	31 (17.7)	<0.001
severe	48 (21.2)	36 (20.6)	
moderate	44 (19.5)	64 (36.6)	
mild	11 (4.9)	24 (13.7)	
none	0 (0)	20 (11.4)	

**Table 3:** Comparative Analysis of Caregiving Experiences: LLRD Children’s Caregivers vs. PKU Children’s Caregivers.

Items	1. Very Bad/ or Never	2. Rather Bad/ or Rarely	3. Neither Good Nor Bad/I Do Not Know/ or Sometimes	4. Rather Good/ or Often	5. Very Good/ or Always	*p*
Impact on Personal Life						
Does caring for an RD child make you experience any of the following emotional states?						
1. emotional control problem						<0.001
LLRD children’s caregivers	7.1%	20.8%	34.9%	30.1%	7.1%	
PKU children’s caregivers	14.8%	33.1%	30.8%	18.3%	2.8%	
2. sense of shame						<0.001
LLRD children’s caregivers	49.5%	27%	14.2%	6.6%	2.6%	
PKU children’s caregivers	77.7%	12%	5.1%	4%	1.1%	
3. nervousness/impulsivity						<0.001
LLRD children’s caregivers	8%	13.7%	36.7%	34.1%	7.5%	
PKU children’s caregivers	12.6%	29.7%	30.3%	23.4%	4%	
4. emotional lability						<0.001
LLRD children’s caregivers	6.2%	13.3%	33.2%	38.9%	8.4%	
PKU children’s caregivers	16%	22.9%	34.3%	22.8%	4%	
5. impatience/irritation						<0.001
LLRD children’s caregivers	5.3%	14.1%	33.2%	38.5%	8.9%	
PKU children’s caregivers	13.1%	21.1%	36.6%	25.1%	4%	
Conflict and Fulfillment of Needs						
6. Has the role of caregiver forced you to give up your own passions, hobbies, and plans?						<0.001
LLRD children’s caregivers	2.6%	13.3%	25.2%	40.3%	18.6%	
PKU children’s caregivers	16.6%	29.7%	22.9%	22.9%	8%	
7. Do you experience conflict between your own needs and those of your RD child?						<0.001
LLRD children’s caregivers	10.2%	23%	30.5%	28.8%	7.5%	
PKU children’s caregivers	37.1%	29.7%	18.8%	9.7%	4.6%	
8. Does caregiving make it difficult to fulfill other roles, i.e., parent/spouse/employee?						<0.001
LLRD children’s caregivers	10.6%	19.5	31%	28.8%	10.2%	
PKU children’s caregivers	32.6%	25.1%	21.7%	14.3%	6.3%	
9. Do you have time to pursue your passions/hobbies?						<0.001
LLRD children’s caregivers	19%	23.9%	35.8%	15.5%	5.7%	
PKU children’s caregivers	25.1%	26.3%	30.3%	13.7%	4.6%	
10. Do you have the feeling that your needs are unimportant to others?						<0.001
LLRD children’s caregivers	8%	16.8%	30.1%	33.6%	11.5%	
PKU children’s caregivers	20%	26.3%	24.6%	19.4%	9.7%	
Physician and Healthcare Support						
11. Support caregivers receive from physicians						<0.001
LLRD children’s caregivers	15.5%	34.5%	13.3%	29.2%	7.5%	
PKU children’s caregivers	6.3%	10.8%	19.4%	46.8%	16.6%	
12. Physicians’ empathy						<0.001
LLRD children’s caregivers	9.3%	28.8%	8.4%	42%	11.5%	
PKU children’s caregivers	4.6%	9.1%	16.6%	52.6%	17.1%	
13. Access to financial help with rehabilitation for RD children						<0.001
LLRD children’s caregivers	35.4%	35%	10.2%	16.8%	2.6%	
PKU children’s caregivers	13.7%	25.1%	39.4%	18.3%	3.4%	
14. Support for RD children and caregivers from healthcare professionals						<0.001
LLRD children’s caregivers	17.3%	35.4%	8.8%	31%	7.5%	
PKU children’s caregivers	6.3%	18.8%	15.4%	48%	11.4%	
15. Physicians’ practical information about RD						<0.001
LLRD children’s caregivers	17.3%	28.3%	9.7%	35.8%	8.9%	
PKU children’s caregivers	3.4%	9.1%	11.4%	46.9	29.1%	
Family Dynamics and Social Support						
16. Do you feel supported by your family and loved ones?						ns
LLRD children’s caregivers	4%	11.1%	19.9%	31%	34%	
PKU children’s caregivers	3.4%	13.7%	15.4%	24.6	42.9%	
17. I can count on practical help from my relatives/friends						ns
LLRD children’s caregivers	26.1%	28.8%	25.2%	10.2%	9.7%	
PKU children’s caregivers	26.8%	24%	23.4%	11.4%	14.3%	
18. I can count on practical help from my family						ns
LLRD children’s caregivers	18.6%	21.7%	25.7%	16.4%	17.7%	
PKU children’s caregivers	22.8%	17.7%	21.1%	17.7%	20.6%	
19. I can count on emotional support from my family						ns
LLRD children’s caregivers	4%	11.1%	19.9%	31%	34%	
PKU children’s caregivers	3.4%	13.7%	15.4%	24.6%	42.9%	
20. I experience conflict in my family						<0.001
LLRD children’s caregivers	31%	21.2%	30.1%	14.6%	3.1%	
PKU children’s caregivers	50.9%	22.3%	16%	9.7%	1.1%	
Financial Challenges and Medication Concerns						
21. Are you worried about your family’s finances?						ns
LLRD children’s caregivers	6.6%	12.8%	32.7%	23.4%	24.3%	
PKU children’s caregivers	6.3%	12%	32%	26.3%	23.4%	
22. Is the cost of medicines and medical care a heavy burden for you?						<0.05
LLRD children’s caregivers	9.3%	14.6%	23.9%	28.8%	23.4%	
PKU children’s caregivers	14.9%	16%	27.4%	26.3%	15.4%	
23. How do you assess your financial situation?						ns
LLRD children’s caregivers	4.9%	18.6%	14.1%	56.2%	6.2%	
PKU children’s caregivers	5.1%	16.6%	20.6%	50.3%	7.4%	
24. Do you experience problems with the reimbursement or purchase of medicines?						<0.05
LLRD children’s caregivers	19.9%	26.5%	13.7%	35.4%	4.4%	
PKU children’s caregivers	6.9%	24%	10.8%	52.6%	5.7%	
25. Contacts with the healthcare system						<0.001
LLRD children’s caregivers	2.6%	16.8%	31.9%	27.8%	20.8%	
PKU children’s caregivers	14.3%	33.7%	26.9%	14.3%	10.8%	

ns—not significant.

## Data Availability

The original contributions presented in the study are included in the article, further inquiries can be directed to the corresponding author.
